# Use and evaluation of a mentoring scheme to promote integration of non-medical prescribing in a clinical context

**DOI:** 10.1186/1472-6920-14-177

**Published:** 2014-08-25

**Authors:** Dianne Bowskill, Oonagh Meade, Joanne S Lymn

**Affiliations:** School of Health Sciences, University of Nottingham, Queens Medical Centre, Nottingham, UK

**Keywords:** Mentoring, Non-medical prescribing, Pharmacology, Prescribing implementation

## Abstract

**Background:**

Growing numbers of non-medical health professionals are attaining prescribing rights through post-registration non-medical prescribing (NMP) courses in the UK. However, not all implement prescribing post-qualification. This study evaluated the uptake and perceived usefulness of a mentoring scheme for two cohorts of NMP students at the University of Nottingham. The scheme paired students with qualified mentors with whom they had an opportunity to discuss the integration of prescribing theory into practice.

**Methods:**

Mentors were allocated on days 2–5 of the course. Surveys were distributed to students who completed the NMP course [n = 63] and their mentors. Likert-scale and open-ended questions addressed: use, perceived usefulness, and positive and negative aspects of the mentoring scheme. Semi-structured interviews were conducted with both students (n = 6) and mentors (n = 3) to explore their experience of the mentoring scheme in more detail. Students were purposively selected for interview depending on their level of use of the mentoring system. Interviews were analysed using thematic analysis.

**Results:**

The response rates were 65.1% (n = 41) and 56.3% (n = 36) for students and mentors respectively. Just over half of students (57.1%) accessed their mentor. Having a sufficient support network was the key reason for not using the scheme. Students found mentors helpful for: moral support (68.2%); contextualising prescribing (71.4%); and helping them to think about implementing prescribing in practice (72.7%). Fewer mentors felt they helped in relation to contextualising (57.9%) or implementing prescribing (31.6%). Less than half the students and mentors surveyed agreed that they received/provided assistance related to the integration of prescribing theory into practice (38.1% and 42.2% respectively) and assistance with assignments (36.3% and 45.5% respectively).

Interviews suggested that students found it difficult to focus on implementing prescribing because of the academic demands of their course, which impacted on uptake and use of the mentoring scheme. Students emphasised the importance of being paired with a prescriber who was successfully prescribing. Mentors benefited from sharing and refreshing their academic knowledge.

**Conclusions:**

Students and mentors derived benefits from participation in this scheme. This intervention may be better as a post-qualification support resource when students are ready to consider their future prescribing practice.

## Background

Recent changes to UK legislation have allowed nurse, pharmacist and other allied health professional (physiotherapists, podiatrists, chiropodists and optometrists) non-medical prescribers the same legal prescribing rights as doctors [1], which means that this group now have the most extensive prescribing authority of all non-medical prescribers worldwide [2]. On successful qualification as a non-medical prescriber, these professionals have both independent and supplementary prescribing rights. Independent prescribing, where the non-medical prescriber is accountable for both diagnosis and prescribing, is the most commonly used form of prescribing for non-medical prescribers [3–6]. Supplementary prescribing (also used by radiographers) where the doctor is responsible for the diagnosis and the non-medical prescriber has the authority to prescribe from a patient-specific clinical management plan. although used less frequently, is still used by some non-medical prescribers for the treatment of complex patients [3], as a result of local NMP policies and as a personal preference [5].

There is an emerging body of evidence that NMP has benefits both for patients and prescribers. Research suggests that patients: are satisfied with non-medical prescribing [7]; feel that this form of prescribing improves both their access to medicines and the efficiency of health services [8]; and find that receiving medication from non-medical prescribers can be less stressful than receiving a medication from a doctor [9]. While there are acknowledged stressors related to taking on this enhanced role for non-medical prescribers [10], there is also evidence that the increased autonomy and ability to provide holistic care increases job satisfaction [10, 11].

Whilst the majority of those qualified go on to prescribe for patients in practice [4, 12] there are a small but significant number who do not prescribe although qualified to do so [3, 13–18]. Unsuccessful implementation is costly and there is a clear expectation from politicians, managers and health professionals that investment in NMP education will result in prescribing activity. Discrepancies in implementation may be accounted for by several organisational factors, such as prescribers moving in to non-prescribing roles (e.g. management) [4, 11, 19], lack of governance and employer support for NMP [19], and practical barriers at a local level such as access to prescribing pads and computer software [3, 19].

However, there are also individual factors which may impact on prescribers’ use of their NMP qualification, such as their experience level [19, 20]. Confidence has been identified as an important issue relating to performing as a non-medical prescriber [11, 21]. Many new prescribers report a lack of confidence in their prescribing knowledge [4, 11, 22] and seek reassurance from doctors before prescribing for a patient [4, 23]. When a new prescriber is unable to integrate prescribing and professional knowledge, they will not use their qualification to prescribe for patients [4, 9, 24]. It has been suggested that knowledge generated and practiced in one context must be re-contextualised to work in a different setting [9, 25].

The NMP course in the U.K. is an intensive, six-month, part-time course which comprises of 26 days of taught education at an accredited higher education institute and 12 days of practice supervised by a Designated Medical Practitioner (DMP) [26–28]. Qualification as an NMP requires students to pass a number of non-compensated elements of assessment including examinations, portfolio assignments, objective structured clinical examinations, medically supervised practice [29] and an evidence based therapeutics poster presentation.

Students often need to focus on achieving academic success during the NMP course [30], which provides little time for considering their future prescribing practice [4]. Additionally, as the NMP curriculum [27, 28] takes a generic approach to prescribing education, it can be difficult for NMP students to create opportunities to contextualise prescribing in their own specific clinical areas [31]. Whilst prescribing courses have multiple aspects of development and competence as laid out by the Nursing and Midwifery Council (NMC) [29], the main focus of the clinical component (medically supervised practice with a DMP) is the development of clinical reasoning and diagnostic decision-making. The challenge for NMP educators, which relates to the aims of the current research, is to ensure that students are best-placed to leave the NMP course with the tools to integrate prescribing theory into practice. Whilst we have successfully introduced a number of learning tools to promote student understanding of core prescribing concepts, these educational interventions have focused on improving theoretical knowledge among students [31–33] and therefore do not address problems students may have with integrating this knowledge into practice.

Recent research into peer mentoring by doctors supports the idea that practice support can help students deal with the problem of changing practice, benefit professional development for the mentor and ultimately improve patient experience [34]. There is also some evidence that a mentoring scheme among nurse prescribers may be desirable [9, 28] and that nurse prescribers are willing to undertake a mentoring role during the NMP programme [35]. Based on the results of a study of medical supervision in non-medical prescribing students, Ahuja [36] noted that some participants discussed the potential value of having a scheme which linked students to qualified non-medical prescribers as a source of support. Similarly, Dawoud et al. [21] suggest that providing input from qualified pharmacist prescribers in to the NMP course for pharmacists would help students understand the experiences of those already in this role. These suggestions echo those of Cooper et al. [37] who suggest that qualified non-medical prescribers should be involved in practical aspects of training NMP students.

The purpose of this study was to evaluate the uptake and perceived usefulness of a mentoring scheme for NMP students, which was initiated in order to help students integrate their theoretical learning within their specific clinical context. Students were paired with qualified non-medical prescribers working in a similar clinical practice area who acted as a mentor, whom they could contact in order to discuss the integration and implementation of theoretical knowledge gained on the course to their clinical practice context. The aims of the research were:

- To describe uptake and use of the mentor scheme from the perspectives of students and mentors.
- To gain an understanding of students’ and mentors’ motivations for, and experiences of, participating (or not participating) in the mentor scheme.

## Methods

### Mentoring scheme description

All students attending the September 2010 and September 2011 non-medical prescribing courses (n = 74) were allocated a qualified prescribing mentor. Mentors were recruited from a database of qualified non-medical prescribers who had undertaken the NMP course at the University of Nottingham since January 2006. All qualified non-medical prescribers on this database were sent information sheets about the nature of the mentoring scheme and a contact sheet to return if they were prepared to act in this capacity.

Potential mentors were invited to attend a briefing session in the School of Health Sciences, in order to provide them with an update on the NMP course curriculum and the potential role of a mentor. Mentors were offered an opportunity to attend 1 of 2 briefing sessions that took place 2 weeks prior to the start of the course. Briefing sessions were run by two of the authors (DB and JL), who lead NMP education at the University. It was made clear to potential mentors at this session that they are not expected to ‘teach’ students but merely to provide support around the effective integration of prescribing into a specialist area of clinical practice. Mentors were asked to provide mentoring for students from the first to the last day of the course. Following the briefing session, mentors were given a 1 week opportunity to decline the role.

Students and mentors were paired by the authors using a sampling matrix [38] with the following criteria: clinical role, employer and geographical location of clinical area. Forty nine mentors were recruited and paired with the 74 students who enrolled on the first day of the course. Some mentors were allocated to more than one student per cohort and some mentors were allocated to students in both cohorts. The reason for this was because of the need to match students with a mentor in a similar clinical area.

### Design

A mixed methods design was adopted. Firstly, a survey was used to gain feedback from students and mentors in relation to their use and perceptions of the mentor scheme. Semi-structured interviews were then conducted to explore, in more depth, experiences of the mentoring scheme from both students’ and mentors’ perspectives.

### Survey method

Student and mentor evaluations of the scheme were collected using two separate surveys.

Surveys were distributed to all students who completed the course (n = 63). All mentors were sent a survey for each of their students that completed the course (n = 63). Some mentors may have completed the survey more than once as they mentored students from two cohorts or because they mentored more than one student in each cohort. Mentors were, however, encouraged to base each individual survey on their experience of each mentor-student pairing that they had.

Surveys were distributed to students on the last taught day of the course and were returned at submission of the final assignment 3 weeks later. Reminders were sent 1 and 2 weeks after the last taught day. Surveys were sent by email attachment to mentors following course completion by students, an email reminder was sent to all mentors after 2 weeks. The surveys were used to collect data on: patterns of use of the mentoring scheme; support offered and gained; and student and mentor perceptions of any advantages and disadvantages of participating in this scheme.

The surveys contained a mixture of fixed response likert scales and open-ended questions. The survey questionnaires were piloted with a group of community matrons who had been allocated a prescribing community matron mentor in an earlier cohort study.

### Interview method

Student and mentor survey participants from the September 2011 cohort were also asked to indicate whether they were willing to take part in an interview to discuss their experience in more detail, and to provide contact details for follow-up if this was the case.

Sampling of students for interview was purposive in nature depending on their use of the mentoring scheme. Students who expressed an interest at the end of the survey in participating in an interview were categorised into low, medium and high users. Low users were defined as those who accessed the mentoring scheme 0–1 times, medium users were those who accessed the scheme 2–3 times and high users were defined as those who used the scheme more than three times. Two students per group were randomly selected and invited to take part in an interview. Recruitment continued until two participants from each group (6 students in total) agreed to participate in the study.

Students were sent information sheets about the interview study prior to participation and were asked to sign a consent form prior to the interview. Interviews were conducted either at students’ workplaces or within the university with a researcher (OM) who was not involved in the provision of NMP education, and was not known to the participants. Interviews were conducted within 12 weeks of survey completion.

Interviews lasted approximately 20–35 minutes and were guided by a semi-structured interview schedule. Questions related to student motivation for using or not using the mentoring scheme, student experience of contacting their mentors to initiate the mentoring relationship, their experience of the mentoring process (including support needs, support offered and any unmet needs) and their overall evaluation of the suitability of the scheme and the particular pairing with their mentors.

Sampling of mentors was convenience in nature. Mentors from the September 2011 cohort who expressed an interest in being interviewed were sent a participant information sheet for the interview component of the study and a consent form. Potential participants were asked to sign and return the consent forms before taking part in an interview, which was conducted over the telephone within 12 weeks of the surveys being completed. The interview schedule contained questions relating to their motivations to become a mentor, their experiences of undertaking this role, and any perceived advantages and disadvantage of the role.

### Data analysis

Quantitative survey responses were analysed using descriptive statistics and qualitative survey responses were analysed using content analysis to examine the prevalence of different response types.

Student and mentor interviews were analysed in two separate analyses using an inductive thematic approach. All interview transcripts were anonymised and initially coded for themes relating to participants’ motivations for, and experiences of, using the mentoring scheme (either as a student or mentor). A preliminary set of themes was developed, which contained a number of higher-order overarching themes and subthemes. This set of themes was developed and amended through careful re-reading of all the interview transcripts, until the themes allowed as full a description of the data as possible.

### Ethical approval

This project received ethical approval from the University of Nottingham Medical School Ethics Committee (Project Code B/06/2010).

## Results

### Survey

#### Response rate

Whilst 74 students were allocated a buddy on the first day of the course, only 63 completed the course and were available to participate in the evaluation. The survey was completed by 41 out of 63 students, representing a response rate of 65.1%. 36 of the 63 surveys sent out to mentors were returned, representing a response rate of 57.1%.

#### Student use and evaluation of the mentoring scheme

Just over half of the student respondents (56.1%, n = 23) reported accessing their mentor and most of these students accessed their mentor three times or less (60.8%, n = 14). Eleven students provided reasons for why they did not access their mentor. The main reason (n = 7) was that students had sufficient support from others including tutors, work colleagues, and student colleagues (e.g. ‘*Work in an area where others have undertaken the course as well as three who are currently on the course. Therefore I felt I had enough support’*). Individual reasons for not accessing their mentor included: difficulty contacting their mentor; poor suitability of their mentor to their practice area; geographical distance as a barrier to meeting; and feeling uncomfortable about seeking support.

Student ratings of the usefulness of the mentoring scheme are presented in Table 1. The majority of respondents agreed or strongly agreed that the scheme was useful (60.6%). Students who used the scheme found it helpful for: receiving moral support (68.2%); contextualising prescribing in their practice area (71.4%); and thinking about implementing prescribing in practice (72.7%). Less than half of the students who used the scheme agreed or strongly agreed that the mentoring scheme was helpful for assignments (36.3%) or for integrating theory from the course into practice (38.1%).

**Table 1:** **Student responses to survey items regarding the usefulness of the mentoring scheme**

	Strongly disagree (%)	Disagree (%)	Neither agree nor disagree (%)	Agree (%)	Strongly agree (%)
*I think the mentoring scheme is useful (n = 33)*	3	3	33.3	27.3	33.3
*I found contact with my mentor useful (n = 23)*	4.3	0	26.1	34.8	34.8
*My mentor helped me with assignments (n = 22)*	13.6	31.8	18.2	13.6	22.7
*My mentor gave me moral support (n = 22)*	4.5	13.6	13.6	27.3	40.9
*My mentor helped contextualise prescribing in my practice area (n = 21)*	4.8	14.3	9.5	33.3	38.1
*My mentor helped me think about implementing prescribing in my practice (n = 22)*	4.5	4.5	18.2	40.9	31.8
*My mentor helped me to integrate theory from the course in practice (n = 21)*	4.8	14.3	42.9	23.8	14.3

#### Mentors evaluation of the scheme

Most mentors agreed or strongly agreed that the mentoring scheme was useful (85.2%), would recommend it to others (74%) and were happy to act as a mentor again (93.4%). All mentors who were contacted by their students felt they were able to provide assistance. A full breakdown of how mentors felt they provided support to their students is included in Table 2. Most mentors felt that they were able to provide moral support (78.7%) and could help their students to contextualise prescribing in practice (57.9%). Less than half of the mentors who had contact with their students felt they helped with: assignments (45.5%); integrating theory into practice (42.2%); and implementing prescribing in practice (31.6%).

**Table 2:** **Mentor responses to survey items regarding how they helped students**

	Strongly disagree (%)	Disagree (%)	Neither agree nor disagree (%)	Agree (%)	Strongly agree (%)
*I was able to help my students with assignments (n = 20)*	20	20	15	20	25
*I gave my student moral support (n = 19)*	5.3	0	15.8	57.6	21.1
*I was able to help my student contextualise prescribing in practice (n = 19)*	15.8	5.3	21.1	26.3	31.6
*I helped my student to implement prescribing in practice (n = 19)*	36.8	21.1	10.5	10.5	21.1
*I was able to help the student integrate theory from the course (n = 19)*	21.1	10.5	26.3	21.1	21.1

Twenty two mentors replied to an open-ended question about the positives of the role. The response categories are summarised in Table 3. The most common benefits included feeling positive about sharing their own experiences to help others and refreshing or updating knowledge and practice.

**Table 3:** **Thematic summary of responses by mentors about the positives of the mentor role**

Positives of the role	n	Example comments
Feeling positive about helping others through sharing experience	10	*Felt pleased to be able to offer help to another student undertaking the course as from experience it can be very tough but the end rewards are worth it.*
Revising/updating own knowledge/practice	4	*Discussion about drugs etc. and accountability helped me to refresh my own knowledge and challenge my practice*
Personal enjoyment	3	*I enjoyed spending time with them both.*
Developing links with other clinical teams	2	*It was good to be able to network and meet people from similar clinical areas to mine.*
Provided structure to support given to colleague	1	*I work in the same office as [name omitted] and would have taken on this role anyway, as I am the first in the team to complete this course, but it probably gave some meetings more structure.*

Eight mentors responded to an open question asking them to detail any negative experiences of the role. The mentoring relationship not working out as planned, or a lack of contact from students was identified as a negative (e.g. ‘*Was disappointed the student didn’t acknowledge my email and I had no further contact from her’)*.

### Interviews

#### Student interviews

Six students took part in the interviews. All student participants are given codes in the extracts below (S1-S6 for students). Two students did not use the mentoring scheme (S1, S2), another had tried to use the scheme but stopped due to limited contact from her mentor (S3) and three students used the scheme (S4, S5, S6). One student (S4) who used the scheme was paired up with a colleague rather than a mentor outside of her practice and therefore her experience may differ from the other students.

In the analysis that follows, key similarities and differences between students’ experiences are highlighted, as the group had some common and some heterogeneous experiences of the scheme. The two over-arching themes were ‘managing course demands – impact on use of mentoring scheme’ and ‘mentor pairing- converging and conflicting preferences’

#### Managing course demands – impact on use of mentoring scheme

This theme describes how students’ use of the mentoring scheme was influenced by their experiences of dealing with the multiple demands they were facing while completing the course. Their concerns about completing the course and managing competing demands on their time influenced their uptake and patterns of use of the mentoring scheme.

#### The immediate need for moral and practical course-related support

The majority of students described how dealing with the demands of the course as well as coping with working life was stressful and an area where they felt they needed support. Some students such as S3 accessed their mentor to seek help with coping with the multiple demands of the course:

*S3: It was right at the beginning of the course when we started going through all the work and stuff and you think god how am I going to do this?*

There was also evidence from students that they received this moral support from their mentors. For example, S6 valued the encouragement she received from her mentor to persist with the course after failing her first attempt at her pharmacology exam:

*S6: She helped me to sort of pick myself up and say right you have got to blast the poster, you have got to do really well on that, get your assignments handed in.*

In addition to moral support, students mentioned seeking advice on aspects relating to completing the course. For example, S5 asked for advice on where to access supervision and how to use that supervision time:

*S5: I wanted to have some guidance as to how she accessed the medical supervision…… and what kind of proportion of time was spent directly with her medical supervisor.*

Students who accessed their mentors also reported gaining support around academic components of the course, such as exam and assignment tips. S4 got support from her mentor in relation to managing expectations about assessments.

*S4: We did talk about things like what to expect from the assessments, the exam, the OSCE, things like that.*

S2 was the only student who felt that she did not need the support of a mentor as she felt adequately supported by the course tutors:

*S2: I felt the support that we got within here, with the tutors and being that I come into [hospital name] a lot anyway I could access the tutors.*

In summary, students who accessed their mentors primarily did so in relation to coping with the demands of completing the course rather than in relation to discussing the integration of prescribing theory in to practice. Only two students described discussing the integration of prescribing theory in practice. S4 discussed how they would begin implementing prescribing in their practice team:

*S4: We have talked…… more how we implement if afterwards, about things we need to do in our team to sort of get prescribing off the ground.*

#### Difficulty finding time to consider prescribing implementation

The students who did not discuss prescribing implementation with their mentors described how they had difficulty finding time to discuss this with mentor. One student who didn’t access a mentor mentioned that she could not find time to fit this in as she was already shadowing other prescribers in practice:

*S2: I managed to find people in the [team type] that had done the prescribing or other consultants that I could spend time with…… I couldn’t have really fitted in anymore.*

This difficulty in finding time to use the scheme was also mentioned by S6.

Three students mentioned that it was not the right time to concentrate on implementing prescribing, due to their need to focus on completing the course. S1 described not having ‘the head space’ to think about it at the time. Similarly, S3 discusses how her needs for support in relation to prescribing implementation will begin to surface once she begins prescribing:

*S3: I think once you start writing prescriptions, then that’s when other problems come up don’t they, that you have not come across until you actually start writing.*

#### Student-mentor pairing- converging and conflicting preferences

Student experiences of the mentoring scheme highlighted both converging and conflicting preferences on what would constitute an ideal student-mentor pairing. These similarities and differences in opinions are drawn out in the subthemes that follow.

#### Valuing mentors’ prescribing experience

One point of agreement mentioned by three students was the value of being paired with a prescriber who was successfully implementing prescribing in practice, as highlighted by S6:

*S6: It was more that spurring on and someone who has been there before and has been successful and continues to prescribe.*

S4 felt she may have experienced additional benefits to the mentoring scheme if her mentor had already started to prescribe:

*S4: She hasn’t prescribed yet and possibly if I was put with someone who is already using it and already prescribing, there might be a dimension they can bring to it.*

#### Benefits of geographical proximity

Two students also discussed how distance from their mentors may have created a barrier to engaging with their mentors. S3 felt that she may have been able to interact better with her mentor if she had been geographically closer:

*S3: I think it would have been better if it had been someone closer to me, we could have probably met up.*

In a related way, S4 valued being in close proximity to her mentor (working in the same clinical area) as this meant she could ask questions as and when questions arose:

*S4: I can just ask her things very ad hoc*

#### Matched clinical practice areas – diverging perspectives

Students also discussed the relative merits and demerits of pairings with mentors who worked within or outside their clinical practice areas and within or outside their team. S4 valued the fact that her mentor was in a similar practice area as she knew they would face similar difficulties in terms of prescribing in practice:

*S4: We are working in the same speciality, we have got the same challenges to face.*

However, S2 felt that it would not have made any difference to her uptake of the scheme if she had been paired with someone who worked within their clinical practice area:

*S2: So even if they had worked in my area I probably still wouldn’t have done, it wouldn’t have been something that I felt I needed to do.*

S3 and S1 both discussed how they felt it was a disadvantage to be paired with a mentor that they did not know from outside their practice team as they felt uncomfortable contacting a stranger. S1 found it difficult to initiate contact with a mentor she didn’t know:

*S1: to ring somebody up that you just don’t know and to be ‘oh my god I’m stressing and’ it would just seem very weird and hard over the phone.*

Three students however, noted advantages of having an objective support source from outside their practice team. S6 felt that it was useful to have someone from outside her practice; both in terms of the type of prescribing practice and the fact that they were located in a different team. She valued the objectivity of being paired with a mentor outside of her practice team:

*S6: It would have been probably too safe to have another specialist nurse in [speciality name]…… it was helpful to have someone who was objective, who didn’t know the team dynamics.*

There appeared to be advantages and disadvantages of being paired with mentors that students knew or that worked in similar practice areas.

### Mentor interviews

Three mentors took part in the interviews. Two mentors (M1 and M2) had two students over the course of two cohorts. The third mentor (M3) volunteered to take on this role for a colleague and as such, this was her only NMP mentoring experience. Her experience was slightly different in that she already worked with her student and therefore their interactions were more informal than the mentoring relationships established by the other two mentors. The other two mentors had experience of mentoring students who had engaged or did not engage with the process. The three over-arching themes which encapsulated mentor experiences were: ‘supporting students with course demands’,  ‘refreshing academic knowledge’ and ‘encouraging prescribing practice within the limits of student readiness’.

#### Supporting students with course demands

**Offering a heightened understanding of student experiences** All mentors expressed a strong desire to share what they had learned and to pass on the benefit of their learning experience to others:

*M3: Having done the course I suppose you feel you want to support other people.*

All three mentors felt they were able to offer students a heightened level of understanding of the particular demands of undertaking the course, based on their personal experience of undertaking the NMP course, as described below by M3:

*M3: Having been through the course I knew the areas that I found difficult…… areas like medical supervision and how you manage to negotiate that with the GPs.*

#### Sharing study support tips

All participants discussed how they offered students advice on assessments and studying tips/strategies as part of the mentoring process. This appeared to be the key function of the mentoring relationship:

*M1: It wasn’t prescribing issues it was more ‘I need help with a particular part of the course’.*

Two mentors mentioned sharing course work examples with their students in order to help them with their study assignments. M2 showed his student his poster as a means of demonstrating what mistakes he made so that his student did not replicate these:

*M2: I was able to show the student my poster…… my student’s initial reaction to that was ‘that’s fantastic’, and I said ‘no it’s not, I scraped through and my poster is a perfect example of what not to do.*

All mentors felt that they could therefore provide the benefit of an experienced opinion on coursework for their students:

*M3: When she did her poster presentation I listened…and gave her feedback.*

#### Providing reassurance

Two mentors also noted how they provided assistance to their students in terms of giving them reassurance or help in planning their academic workload. M1 wanted to reassure her student that they were coping well in the face of the multiple academic demands they were experiencing:

*M1: You can get very bogged down and a lot of things needing to be done at the same time. Sometimes I made them sit down, plan it better, and reassure them they’re doing okay.*

#### Refreshing academic knowledge

All mentors felt that undertaking this role was beneficial for them. Two of the mentors had mentored two different students over consecutive cohorts and discussed how they had varying levels of contact from their students. They described how not having any contact from their student, while not a disadvantage of the scheme, was disappointing and they benefited more from mentoring students who engaged in the mentoring scheme. M2 describes how having more contact from his second student was of benefit for him and his student:

*M2: He pretty much got in touch with me within a few weeks of starting the course…… which wasn’t just of benefit to him but you know, of benefit to me also.*

He later explains how this contact was particularly helpful for refreshing his own knowledge on drugs that he would not ordinarily prescribe in his practice setting:

*M2: It reminded me of things that I would perhaps otherwise have forgotten you know, in relation to specific drugs that I wouldn’t ordinarily prescribe.*

#### Encouraging prescribing development according to student readiness

**Sharing prescribing practice experience to develop student confidence** All mentors shared their prescribing practice experiences in different ways to help with their students’ confidence in prescribing. Two mentors described how they reassured their students of the benefits of integrating prescribing into their practice post-qualification. M3 discussed how she assured her student that her job would become easier to manage when she is able to prescribe in practice:

*M3: I said “it is easier when you can just do it [writing prescriptions] yourself.”*

Mentors also offered students the opportunity to shadow their clinical work in different prescribing scenarios in order to develop their practical experience of prescribing. M3 offered shadowing experiences to her student and promoted discussions of prescribing scenarios:

*M3: I mean (student name) came and sat with me for two mornings…… and I asked her what she might do and so talking through scenarios.*

#### The importance of appropriate clinical matching between students and mentors

In terms of their ability to offer help in relation to prescribing, all three mentors discussed the value of having an appropriate pairing with their students, in terms of their clinical practice areas. One of the reasons for this was perceived differences in the medications that would be prescribed across different practice settings:

*M3: I wouldn’t have thought buddy me up with somebody who was working in [speciality name] would particularly work well because the medications that we would prescribe and the contexts in which we would be prescribing would be completely different.*

M1 describes how she feels that clinical areas may not matter so much for the purposes of helping students with the academic component of the course, but that in terms of developing prescribing competence, a matched clinical area would be very beneficial:

*M1: If it’s just the practicalities of the course it doesn’t matter what you’re doing, if there’s any prescribing issues, if it was something that I had no knowledge about and no competence about at all then it would have been a waste of time.*

#### The limits of student readiness to consider prescribing

While mentors expressed a willingness to help develop students’ prescribing practice, as well as providing academic support and reassurance, they discussed difficulties with providing this type of assistance while students were undertaking the NMP course. All three mentors discussed how the timing was inappropriate in terms of being able to help students in this capacity. Two mentors discussed the fact that students were too busy with the demands of the course, to focus on prescribing, as illustrated in the quote below:

*M2: He was always of the opinion let’s not run before I can walk, let’s get through the course first and then I’ll give it some thought.*

Similarly, one mentor felt it was difficult to offer advice on implementing prescribing before students get their prescribing rights. She would like to offer support after the academic NMP course once students get their prescription pads to help them achieve the vital endpoint of prescribing in practice:

*M3: Well, I think I would offer support for them when they first got their prescription pads…… the purpose of doing the prescribing [course] is to be able to prescribe.*

## Discussion

Mentoring, as distinguished from educational supervision, appraisal or other summative processes, provides one-to-one support for those professionals who are newly qualified or undergoing a professional transition [39]. Whilst there is a growing body of literature which reports the use of mentoring schemes in the medical arena [40–42] and despite the Department of Health recommendations for mentoring of NMP students [28] the current study is the first to describe the use and evaluation of such a scheme.

The original intention of the scheme, which paired NMP students with experienced non-medical prescribers, was to support students to reach the vital endpoint of using their prescribing qualification to implement prescribing successfully post-qualification. In this respect this scheme was only partly successful with both students and mentors reporting that alongside contextualising prescribing, moral support to get through the course, was a key form of support both received and offered.

Interview data from both students and mentors discussed how, due to the academic challenges of the course, students found it difficult to focus on integrating, or contextualising, their course knowledge into practice. Typically students undertaking the NMP course hold senior roles and have little or no access to backfill arrangements whilst undertaking their studies. These are mature students with family responsibilities and busy lives. Undertaking this course can be a significant challenge and is therefore understandable that students may not have been fully committed to thinking about integrating prescribing theory into practice whilst undertaking the course. It may also explain why mentors may have felt more able to provide moral support and academic experience than help with contextualising, implementing or integrating prescribing theory into practice and why students highly valued the moral support they received.

Whilst the scheme may not have been used by all students, or may not have been used entirely for the purposes for which it was originally intended, benefits for both students and mentors were evident. Benefits of mentoring schemes for mentors have been recognised by a number of previous studies conducted with doctors [39, 40, 43, 44] and include sharing of experiences, a sense of satisfaction and encouraging mentors to maintain currency in the relevant clinical area [39]. Developing senior doctors as mentors for more junior staff has also been suggested to be a form of continuing professional development for both parties [44]. This medical literature compares well with the results of this study where mentors felt that taking on this role acted to refresh their own knowledge. Data from a recent national survey of nurse prescribers has reported that a third felt that the continuing professional development opportunities afforded to them were not adequate to maintain patient safety [45]. Whilst this suggests that there has been an improvement in the continuing professional development opportunities for nurse prescribers since the previous national survey [46], there remain ongoing issues in this area. This may be compounded by the fact that many NMP leads within organisations do not themselves have a prescribing qualification [47] and may not therefore be able to effectively understand the requirements of these professionals in relation to continuing professional development.

All students who were interviewed mentioned the importance of being paired with a prescriber who was successfully prescribing in practice. Therefore, arranging this type of contact for students may be important to help them realise that successful implementation of prescribing can be achieved. Some students may already have significant academic, peer and colleague support, but mentors may still be helpful in terms of demonstrating to students that prescribing can be achieved. Indeed it has been suggested that in order for mentees to learn about the environment they are entering in a risk-free manner, mentors should not be the mentee’s line manager at work or involved in their appraisal in any way [39].

A recommendation from this study is the potential importance of a similar mentoring scheme being implemented post-qualification for new prescribers. There has already been a call for the development of mentorship schemes for newly qualified prescribers [11]. In particular it has been suggested that those working in speciality areas where prescribing practice is less well-defined may benefit from mentorship from prescribers with more experience [23]. Similarly, for newly qualified prescribers who are not already working alongside a team of qualified non-medical prescribers who can support them with implementation, this type of scheme may be useful. Dawoud [21] described the first six months of prescribing practice for pharmacists as a ‘blind alley’ (p.50). Similarly, Bowskill et al. [4] reported that new prescribers often engage in permission seeking from doctors whilst prescribing initially, which subsides after approximately a year. Therefore, a mentoring scheme may be particularly important when non-medical prescribers begin their post-qualification prescribing practice. Healthcare organisations could potentially facilitate this type of scheme by creating a register of qualified non-medical prescribers who would be willing to undertake this role for newly-qualified colleagues. In this study, almost all mentors agreed that they would act as a mentor again which indicates that non-medical prescribers are keen to be involved in such schemes, even when they are giving their time voluntarily.

A further lesson learned from the current study, which would be relevant to the design of future mentoring schemes in NMP, is that students had different preferences in terms of being matched with qualified prescribers who were either working within or outside their current work team or practice area. Further research would be needed to clarify what preferences newly qualified prescribers would have in terms of a suitable pairing so that the potential benefits of this scheme could be maximised. Similarly, providing newly qualified non-medical prescribers with the option to choose their mentor may also be beneficial.

This study was a valuable initial exploration of the potential of a mentoring scheme to help NMP students reflect on the integration of theoretical prescribing knowledge in to their clinical practice. This project provided an important description of uptake, patterns of use, and student and mentor experiences of the scheme. The study, however, is descriptive in nature and based on a pilot study of one mentoring scheme on one NMP course in the U.K. A further potential limitation of this work is that some mentor participants may have completed the survey more than once. While mentors were asked to base their responses on each student-mentor pairing, it is possible that some participants’ views are over-represented in the data. This could be remedied in future work by assigning only one mentor per student.

Further research needs to be conducted to examine whether such a scheme could improve outcomes related to newly-qualified professionals’ confidence in implementing prescribing and their prescribing activity. This would be especially relevant if a scheme were to be implemented by healthcare organisations to examine the costs and benefits experienced in the implementation of a mentor scheme. NMP provision in the U.K. is more widespread and developed than other countries worldwide. Therefore, any lessons learned in the U.K. in terms of enhancing NMP education and post-qualification support are important internationally for those seeking to deliver NMP training courses and/or those tasked with embedding non-medical prescribing within healthcare service provision.

## Conclusions

Students and mentors both derived benefits from participation in this scheme. The benefits for students related to both the intended aim of the scheme (assistance with integrating prescribing theory and practice) and the practical and emotional support offered/received in relation to course completion. Some students and mentors reported that students were not ready to consider their prescribing practice whilst completing the course, which shaped their patterns of use, and how they used the scheme. Mentors reported benefits in terms of ensuring currency of knowledge suggesting that it may also act as a suitable form of continuing professional development. These data suggest that a mentoring system for students undertaking the course is an effective mechanism of support. Similar mentoring systems should be available to support newly qualified non-medical prescribers to ensure that they implement prescribing in practice. The results of this study will be useful to educators, employers and policy makers involved in developing the NMP context nationally and internationally.
